# Nitrous Oxide Gas in Surgical Operations

**Published:** 1866-02

**Authors:** P. W. Ellsworth

**Affiliations:** Hartford, Conn.


### NITROUS OXIDE GAS IN SURGICAL OPERATIONS.

EDITOR MEDICAL AND SURGICAL REPORTER:—In your number for January 6th, 1866, is a letter from the distinguished surgeon J. M. CARNOCHAN, of New York, respecting the use of nitrous oxyde gas in operations. In this he states:

"This is the first capital operation performed under the influence of the gas since the great discovery of WELLS, of Hartford, 22 years ago," etc.

By turning to pages 52 to 68 of the work called Anæsthesia, published in 1859, by HON. TRUMAN SMITH, in defense of WELLS before Congress, you will find a full account of an amputation performed by me, in 1848, 18 years ago—with a success fully equal to that attained since, either by gas or chloroform. *This* was the first amputation. The same reasons are there given for preferring the gas to other anæsthetics, which are urged by Dr. CARNOCHAN. Probably he was not aware of this operation when he made the above statement.

In that case WELLS himself gave the gas.

Very respectfully yours,

P. W. ELLSWORTH.

*Hartford, Conn., Jan.* 12, 1866.

